# Probiotics as a Weapon in the Fight Against COVID-19

**DOI:** 10.3389/fnut.2020.614986

**Published:** 2020-12-15

**Authors:** Elisavet Stavropoulou, Eugenia Bezirtzoglou

**Affiliations:** ^1^Department of Medicine, Centre Hospitalier Universitaire Vaudois (CHUV), Lausanne, Switzerland; ^2^Service of Infectious Diseases, Central Institute of Valais Hospitals, Sion, Switzerland; ^3^Department of Medicine, Lausanne University Hospital and University of Lausanne, Lausanne, Switzerland; ^4^Laboratory of Hygiene and Environmental Protection, Medical School, Democritus University of Thrace, Alexandroupolis, Greece

**Keywords:** COVID-19, probiotics, immunology, coronaviruses (CoV), vanA, adjuvant, therapy, protein E

In our previously published work, we support that probiotics could be used as an adjunctive treatment against COVID-19 (1) and other colleagues have also focused their attention on this subject (2, 3).

Probiotics boost the immune system, enhance the mucosal barrier function and inhibit bacterial adherence and invasion capacity in the intestinal epithelium by being in a direct antagonism with pathogenic bacteria (1). The gut-lung axis is involved in the pathogenicity of bacterial and viral infections, as the intestinal microbiota boosts the alveolar macrophage activity, thus having a protective role in host defense against pneumonia (1). Along these lines, current clinical evidence connects gut, lung, and brain as an entity with communication mediated through complex neural, immunologic inflammatory, and neuroendocrine networks, the so called gut-brain-lung axis (4). There are indications in animals and humans that intestinal microbiota provides bacteria to the lungs, as abundance of *Bacteroides* sp. is observed in the lung following sepsis (5). Moreover, following sepsis, neurologic and cognitive outcomes are observed (4, 6). Without any doubt, the importance of the gut microbiome is stated (1). The composition of the gut microbiome may be used as a predictive tool of disease development and infection severity (1, 7, 8).

Pattern recognition receptors (PRRs) are of major importance for the developing of the innate immune response (9). Probiotics regulate the innate immune cells via interactions between cell wall components or metabolites with host PRRs (10). Yet, probiotic bacteria are activating Dendritic Cells (DCs) and macrophages boosting adaptive immune responses (B cell differentiation, T cell homing, Th17 cell stimulation) (11). The expression of Pattern Recognition Receptors (PRRs) is inflamed in the lung cells during inflammation processes. In this context, macrophages, monocytes and neutrophils are responding by increasing levels of PAMPs (Pathogen-Associated Molecular patterns) and DAMPs (Danger-Associated Molecular Patterns) (12).

Besides that, the PRRs recognize DAMPs (Danger-Associated Molecular Patterns) as danger signals emitted by damaged or necrotic host cells which stimulate the pro-inflammatory response (6). Intruder's viral pathogens show a distinctive particular image of PAMPs giving a specific immune response (9). PAMPs are nucleic acids or glycoproteins indispensable for the pathogens' survival. PAMPs should be identified by PRRs leading to the expression of co-stimulatory molecules such as cytokines and chemokines which, in their turn, should activate the antigen presenting cells and specific adaptive immunity by eliminating pathogens (6, 13). It is of note that the most studied PRRs for pathogens' recognition are TLRs (Toll Like Receptors) which are membrane glycoproteins (12). The expression of Pattern Recognition Receptors (PRRs) is inflamed in the lung cells during inflammation processes. TLR4 signaling in pulmonary stromal cells is critical for inflammation in the airways and probiotics reduce inflammation by limiting the expression of TLR4 (11).

SARS-CoV-2 belongs to the Coronaviridae which is a family of enveloped, positive-sense, single-stranded RNA (+ssRNA) viruses (14, 15). SARS-CoVs possess 3 main structural proteins in the virion envelope (15, 16): (a) protein S (Spike) which permits adherence and fusion, (b) protein M (Membrane) being the most abundant in the virus envelope and participating in the virion assembly together with protein E, and finally (c) protein E (envelope) which is the less abundant protein in the virion envelope possessing a single hydrophobic domain (HD) (16). In addition, SARS-CoV-2 contains the nucleocapsid protein (N) (14).

It is believed that SARS-CoV E protein assembles into a homopentamer (15). Furtherly, SARS-CoV E can oligomerize and form a channel. Particularly, SARS-CoV E proteins create cation-selective ion channels, forming a viroporin (17, 18). It is a proteolipidic pore with negatively charged planar lipids in bilayers which amplify ion conductance and cation selectivity (19).

It is reported that viral ion channels (viroporins) instigate a leakage in host cellular membranes influencing the membrane role (19). Thus, viroporins set out a role of markers of a viral infection (20). Coronaviruses usually possess three types of ion channels: E, 8a and 3a (21). The 3a ion channel has 3 TMD (Trans-Membrane Domain), while the E and 8a ion channels have one TMD (21). Yet, 3a and protein E were found to possess a common structural aminoacid PDZ domain-Binding Motif (PBM) which is known to play a key role in anchoring receptor proteins in the membrane (22, 23). There is evidence that the last four aminoacids in the C-terminus PBM of the E protein are actively participating in the development of SARS-CoV disease resulting in the overexpression of cytokines, called the “cytokine storm” (23).

There has been some evidence that protein E is profusely expressed in the infected cell as it is found mainly in ERGIC (Endoplasmic Reticulum-Golgi Intermediate Compartment), while only a small protein fragment is found in the envelope of the virion (24–26).

It should be reported that the PBM is found only in several coronaviruses, specifically in the genera α and β, while it is not present in the γ genera (24). Without any doubt, it should be interesting to investigate the role of the interaction patterns and whether they interact specifically with the protein E of SARS-CoV or also with protein E from other coronavirus species of the same genus (27).

Moreover, in coronaviruses, the ERGIC intercedes in between the endoplasmic reticulum and the Golgi for dispensing the virions from the infected cell (26) and accommodates Ca2+ transport (28). As a result, activation of the NLRP3 inflammasome occurs going along with an overproduction of interleukin 1β (29). E protein found in the ERGIC participates in the formation of the infectious virion SARS-CoV E protein involves a triple role; viral assembly, virion release, and viral pathogenesis (28, 29). SARS-Cov E protein has been mentioned to connect to five host proteins (Bcl-xL, PALS1, syntenin, sodium/potassium (Na+/K+) ATPase α-1 subunit, and stomatin) (24) in SARS-1, permitting virus replication in the host.

Studies have shown that administration of a p38 MAPK inhibitor boosted the survival rate in mice following SARS-CoV infection (30) defining its importance in SARS-CoV virulence. Thus, the PBM virulent domain of E protein activates the immunopathological mechanisms by using syntenin as a mediator of p38 MAPK induced inflammation (31).

*Lactobacillus* contains a HSP27-inducible polyphosphate (poly P) fraction. Probiotic-derived polyphosphates, strengthen the epithelial barrier function and keep intestinal homeostasis through the integrin-p38 MAPK pathway (32).

The importance of protein E in the pathogenesis of mutant SARS-CoV viruses is stated (18, 19) as mutant of SARS-CoV with inactive E protein in experimental mice model and *in vitro* cell culture able to retrograde protein E in its initial active status (24). Further to the above, the absence of protein E viroporin is related to shrinking of pulmonary edema as well as to lower mortality rates in mice (33). In this vein, other authors observed the absence of protein E to be related to inefficient viral maturation, incompetent progenitors and reduced viral levels (34). Minor PBM mutations of protein E seems to be bearable while PBM main domain remains intact (24). In this regard, knowledge on PBM mutants and their interaction capacity with host cell proteins will provide us information on the virus pathogenicity.

It is known that SARS-CoV protein M is participating also in the viral envelope assembly and release of the virions (35) but in spite of that there is no evidence of protein M role in membrane morphology (35). Membrane deviating morphology seems to be exclusively related to the protein E in SARS-CoV (35).

Moreover, analysis of viral proteins by VaxiJen software in order to test the probability of antigenic proteins showed evidence that protein E was the most antigenic (36).

Here we report the Avian infectious bronchitis virus (IBV) which prerequisites grounds in the hydrophobic domain (HD) of protein E (17). There is biochemical evidence of 2 different oligomeric forms of protein E in IBV; the one is involved in the IBV assembly and the other form participates in the disarray of the secretory pathway (17). It is then expected that broader knowledge of the SARS-CoV protein E will supply us with novel therapeutic targets (37).

Successfully, antiviral drugs that target viroporins were designed (36). Antiviral drugs either inhibit proton conduction in the channel and directly block the channel passage or bind to six sites located outside the channel cavity and inhibit cation conduction with an allosteric mechanism (38). In this context, amantadine and rimantadine were proposed for the treatment of influenza viruses (39).

As stated previously, evidence of additional interaction partners for SARS-CoV E protein could offer a real targeted therapeutic option for the disease treatment (24). Probiotics interact with protein E and result in the internalization of the virion stimulating macrophages, dendritic and mast cells thus, releasing cytokines and chemokines (24) and stimulating the host immunity (40).

Despite the meaningful research progress on viroporins' structure and on the role of SARS-CoV protein E in viral replication, such as assembly, membrane functioning, virion release, inflammation, autophagy and apoptosis, major challenges lie ahead in research in order to get knowledge on the implicated physio-pathological mechanisms and settle therapeutic approaches for the treatment of COVID-19. Medications deactivating the enzymes implicated in these mechanisms should be hopeful as potential antiviral treatments.

Current research is focused on the use of probiotics as a carrier of exogenous antigens into the mucosa, where they can boost IgA production and develop an enhanced T cell response (41–43). In this vein, *Lactobacillus* sp. were used to release influenza H9N2 hemagglutinin or even H1N1 M2e protein as an approach to enhance the influenza virus vaccine efficacy (42–44). Another study reported that oral administration of probiotics can differentially affect gene expression, and may boost the antiviral activity (45, 46).

All the above, suggest the potential effect of probiotics as a potential therapeutic approach for COVID-19.

Nevertheless, probiotics should be used with caution, especially in critically ill patients (1). They are not recommended in immunocompromised patients or those with prosthetic valves due to their potential of invasive infections (1). Moreover, some of them have been incriminated as potential recipients of resistance genes (*vanA* gene cluster due to *E. faecium* SF68 strain) (47). Finally, some have high enzymatic activity, expressing CYP enzymes, specifically P450 that could interfere in drugs' metabolism and bioavailability (48, 49).

Yet, a clearer understanding of the implicated mechanisms of action of probiotics, specification of the biochemical profile of the enigmatic SARS protein E and clinical trials are needed, in order to assign a probiotic as efficient in the prophylaxis or field therapy of COVID-19.

## Author Contributions

All authors listed have made a substantial, direct and intellectual contribution to the work, and approved it for publication.

## Conflict of Interest

The authors declare that the research was conducted in the absence of any commercial or financial relationships that could be construed as a potential conflict of interest.
